# Correlation between vestibular test results and self-reported psychological complaints of patients with vestibular symptoms

**DOI:** 10.1590/S1808-86942012000100010

**Published:** 2015-10-20

**Authors:** Léia Gonçalves Gurgel, Michelle Ramos Dourado, Taís de Campos Moreira, Adriana Jung Serafini, Isabela Hoffmeister Menegotto, Caroline Tozzi Reppold, Cristina Loureiro Chaves Soldera

**Affiliations:** a Graduate level (Speech therapist, master's degree student in the Health Science Graduate Program, UFCSPA); b Graduate level (Speech therapist, UFCSPA); c Master's degree (Speech therapist, supervisor of the VIVAVOZ unit, UFCSPA); d Doctoral degree (Psychologist, adjunct professor, UFCSPA); e Doctoral degree (Speech therapist, adjunct professor, Speech Therapy Department, UFCSPA); f Doctoral degree (Psychologist, adjunct professor, UFCSPA. Productivity bursary, CNPq); g Master's degree (Speech therapist, assistant professor, Speech Therapy Department, UFCSPA, doctoral student in biomedical gerontology, IGG/ PUCRS.). Porto Alegre Federal University – Health Science.

**Keywords:** electronystagmography, speech, language and hearing sciences, psychopathology, vertigo

## Abstract

Cognitive and emotional factors may affect balance; psychiatric conditions are a common component in patient dizziness. The treatment of patients with vertigo may be affected to a greater degree by the suffering due to this disease than by the severity of organic changes.

**Objective:**

This study aimed to investigate associations between vestibular test results and self-reported psychological complaints in patients evaluated during 2009 in an audiology unit at a hospital in Porto Alegre.

**Methods:**

We conducted a retrospective, descriptive-exploratory study of data taken from a database of the software VecWin^®^ and VecWin^®^ 2, developed by Neurograff^®^. We investigated vestibular test results, reports of psychological symptoms reported spontaneously, and information such as age, sex and the presence of vertigo and/or dizziness. This study consisted of three steps: clustering, exclusion/inclusion and quantification.

**Conclusion:**

Age and gender and the presence or absence of vertigo and/or dizziness were not variables that influenced the outcomes of vestibular testing. There was a significant association between the presence of self-reported psychological complaints and normal vestibular test results. Thus, it is crucial that professionals pay attention to psychological issues reported by patients when the vestibular history is taken.

## INTRODUCTION

Otoneurology is the study of the auditory and vestibular systems and their relationship with the central nervous system (CNS)^1^. Vestibulometry is a set of procedures for evaluating bodily balance. Computed vectoelectronystagmography (VENG) is a step in vestibulometry; it is a method for recording ocular movements that are directly or indirectly related with vestibular function^2^. VENG establishes the direction of nystagmus and calculates the velocity of its slow component, a fundamental parameter for assessing the function of the labyrinth^2^.

Vestibular disorders significantly hamper individuals; they may require assistance for even the simplest tasks that previously were part of their normal lives^3^. Vestibular disorders include peripheral vestibular diseases (comprising diseases of the inner ear and/or the vestibular branch of the eight cranial nerve) and central vestibular diseases (involving structures, nuclei, pathways, and vestibular interrelationships within the central nervous system)^4^.

Manifestations of vestibular diseases include dizziness and/or unbalance, which affect about 10% of the world population^3^. Dizziness and unbalance are considered the most common complaints after 65 years of age and comprise 5% to 10% of medical visits per year^3^. One of the reasons that dizziness is such a common symptom is its diverse etiology, which may include diseases of the labyrinth, cardiovascular conditions, neurological diseases (multiple sclerosis, tumors, epilepsy, ischemic attacks, and drug intoxication), and psychogenic factors^5^, ^6^. The physical and emotional conditions that cause dizziness may result in severe loss of function that compromise work, social relations, and domestic activities of patients^3^. Often, vestibular disorders are accompanied by hearing loss and neurovegetative disorders such as nausea, vomiting, intense sweating, and pallor^1^.

The relationship between anxiety and balance is based on the premise that balance disorders and anxiety share central neural circuits with monoaminergic components. These circuits are centered on a network in the parabranchial nucleus, which is a point at which the vestibular system and the area for processing visceral information converge; this network also involves symptoms of avoidance, anxiety, and fear^7^.

It should be noted that a complain t is the first moment of contact between a patient and a healthcare professional; this moment concerns the manifest and conscious contents of the symptom. An initial interview for examining the vestibular system should offer a space for listening and care, so that the patient may speak not only of the symptom, but of his or her suffering as related to it^8^. Therapy may be more influenced by the suffering and behavior of the disease than by the severity of organic findings^3^. Thus, this study is relevant because a high proportion of otoneurologic patients present psychological disorders^3^. The purpose of this study was to investigate associations between the results of vestibular testing and self-reported psychological symptoms in individuals seen at an audiology unit in a hospital in the city of Porto Alegre, RS, from January to December 2009.

## MATERIAL AND METHODS

A retrospective and descriptive-exploratory study was made of the available data of a sample consisting of 304 subjects; data were gathered from January to July 2010. The institutional review boards of the participating entities approved this study (numbers 341/10 and 10/597). The work consisted of studying a database, which did not require subjects to sign a free informed consent form. The institutional review boards authorized the researchers to divulge the data related to the study sample.

Data was gathered by consulting the databases of the *VecWin*^®^ and *VecWin*^®^ *2* softwares, which are used in recording and analyzing vectoelectronystagmography tests at our institution. The computed vectoelectronystagmography unit was a Neurograff^®^ Eletromedicina Ind. & Com. Ltd. device, which has its own software (*VecWin*^®^) and a light bar for presenting visual stimuli^9^. The *VecWin*^®^ software is stored in the computer memory and records clinical history data as well as the vestibular-ocular-motors reflexes resulting from visual stimulation^10^.

The same speech therapist gathered the clinical histories and carried out the tests, which were recorded in the *VecWin*^®^ or *VecWin*^®^ *2* softwares. Patients were not asked directly about psychological complaints; when these were made, they were done so spontaneously; the examiner recorded these complaints when they were offered at any point during history taking or testing. For this study, age, sex, complaint of dizziness and/or vertigo, self-reported psychological complaints, and vestibular test results were recorded.

The study consisted of three steps: gathering and grouping, inclusion/exclusion, and analysis/quantification. At first, data recorded in the software was gathered and grouped into five categories: age, sex, complaint of dizziness and/or vertigo, self-reported psychological complaints, and results of vestibular testing. Psychological complaints were classified based on a previously published method and according to the words of patients themselves, as follows: anguish, depression, fear, anxiety, and memory disorders^11^. Other parameters were added after analyzing the database, namely: stress^12^, lasitude^12^, irritability^12^, panic^13^, insecurity^14^, and discomfort^15^; this depended on whether these self-reported complaints were present in the study sample. The latter were established by applying Bardin's analysis^16^, which refers to content analysis (study of figures of speech, reticence, between the lines content, manifest content, etc.) of the discourse. These are a set of communications analysis techniques that take into account human subjectivity and the meanings that research subjects attribute to content. The aim to is apply systematic and objective procedures to gather quantitative or nonquantitative measures to infer knowledge about the status of message production/reception (inferred variables)^16^.

Secondly, data of all subjects (315 individuals) that underwent vestibular testing from January to December 2009 were included. Eleven of these subjects were excluded from the study because their tests were inconclusive or the clinical history was incomplete. The resulting sample comprised 304 subjects.

In the last step, a statistical analysis was made of the data; the software for this purpose was the Statistical Package for the Social Science (SPSS) version 18.0. The chi-square test was applied to evaluate associations among the results of vestibular testing and the age group, sex, complaint of vertigo and/or dizziness, and self-reported psychological complaints; the significance level was 5% (p = 0.05). Abnormal vestibular tests were those that yielded the following results: deficient peripheral vestibular syndrome (DVPS), irritative peripheral vestibular syndrome (IVPS), including patients in which the only abnormality was a positive result to positioning tests, or the central vestibular syndrome (CVS).

## RESULTS

Of 304 subjects enrolled in this study, 23% were aged between 41 and 50 years. The mean age of participants was 50.77 years.

Female subjects comprised 72.7% of the sample. The complaint dizziness/vertigo was reported by 93.8% of the study sample; however, only 12.5% of subjects spontaneously reported psychological complaints. Table 1 shows that although 94.7% of subjects that spontaneously reported psychological complaints mentioned dizziness and/or vertigo, there was no significant association among these findings *(p*>0.05) (Table 1). Nineteen patients did not report dizziness/vertigo; they underwent vestibular testing because of other auditory complaints, such as tinnitus e hearing loss.

**Table 1:** Association between self-reported psychological complaints and vertigo/dizziness (Chi-square test).

Vertigo/dizziness					
		Yes n (%)	No n (%)	Total n (%)	*p*
Self-reported psychological complaint	Yes n (%)	36 (11.85%)	2 (0.65%)	38 (12.5%)	0.568
	No n (%)	249 (81.9%)	17 (5.6%)	266 (87.5%)	

Total n (%)		285 (93.75%)	19 (6.25%)	304 (100%)	

n- number of subjects; *p*-statistical value of the test

Of 304 subjects, 61.3% had abnormal vestibular tests. Table 2 shows the association between the result of vestibular testing and gender. The vestibular test was normal in 38% of females and 40% of males; there was no significant association between sex and test results (*p*>0.05). Ninety-seven of 187 subjects with abnormal vestibular test were aged over 50 years. Table 3 shows that there was no significant association between age and the results of vestibular testing (*p*>0.05).

**Table 2:** Association between the result of vestibular testing and sex in the sample (Chi-square test).

Result of the vestibular test	Abnormal n (%)	Normal n (%)	Total n (%)	*p*
Sex				
Female	137 (62%)	84 (38%)	221 (100%)	0.440
Male	50 (60%)	33 (40%)	83 (100%)	

n- number of subjects; *p*-statistical value of the test;

* statistically significant association.

**Table 3:** Association between the result of the vestibular test and age in the sample (Chi-square test).

Result of the vestibular test	Abnormal n (%)	Normal n (%)	Total n (%)	*p*
Age				
Less than 49 years	90 (58.8%)	63 (41.2%)	153 (100%)	0.197
Over 50 years	97 (64.2%)	54 (35.8%)	151 (100%)	

n- number of subjects; *p*-statistical value of the test;

* statistically significant association.

Of 285 subjects that reported dizziness and/or vertigo, 177 had abnormal vestibular tests and 108 tested normal. Again, there was no significant association between the presence of dizziness and/or vertigo and the result of the vestibular test (*p*>0.05) (Table 4).

**Table 4:** Association between the result of the vestibular test and the presence of vertigo/dizziness in the sample (Chi-square test).

Result of the vestibular test	Abnormal n (%)	Normal n (%)	Total n (%)	*p*
Vertigo and/or Dizziness				
Yes	177 (62.1%)	108 (37.9%)	285 (100%)	0.278
No	10 (52.6%)	9 (47.4%)	19 (100%)	

n- number of subjects; *p*-statistical value of the test;

* statistically significant association.

Table 5 shows that 55.3% of subjects that spontaneously reported psychological complaints had normal vestibular tests. In this case there was a significant association between the presence of self-reported psychological complaints and a normal vestibular test (*p*=0.019).

**Table 5:** Association between the result of the vestibular test and the presence of self-reported psychological complaints in the sample (Chi-square test).

Result of the vestibular test	Abnormal n (%)	Normal n (%)	Total n (%)	*p*
Self-reported psychological complaints				
Yes	17 (44.7%)	21 (55.3%)	38 (100%)	0.019*
No	170 (63.9%)	96 (36.1%)	266 (100%)	

n- number of subjects; *p*-statistical value of the test;

* - statistically significant association.

## DISCUSSION

Studies that have related vestibular diseases with psychological complaints have noted that most such patients are aged from 41 to 50 years^17^, ^18^, ^19^; our data corroborates these findings. The estimated prevalence of balance disorders and vertigo comprises 5% to 10% of medical visits per year, and affect 40% of people aged more than 40 years^20^. There is a strong correlation between vertigo and psychological disorders among adults and elderly individuals^3^, ^21^, ^22^.

Other studies have shown that women are more susceptible to otoneurological conditions than men; our sample in which 72.7% were female was similar in this regard to previously published papers^2^, ^18^, ^23^. It is said that the prevalence of dizziness is higher in women, reaching a 2:1 ratio^24^. The presence of self-reported negative emotional states (especially stress) may be associated with changes of the dynamic balance in young adults, irrespective of sex and age^25^.

We found that 94.7% of subjects that spontaneously reported psychological symptoms also complained of dizziness and/or vertigo. Other published studies have shown that psychological symptoms occur together with vertigo and/or dizziness in 56.38% of subjects even when psychological symptoms are formally part of an evaluation protocol; in decreasing order they are anguish, anxiety, fear, depression, and memory disorders^11^. Psychic symptoms appear to be a current representation of past conflicts that were experienced traumatically and that may be reactivated, thereby explaining the onset of vertigo^11^.

A normal vestibular test associated with a complaint of dizziness and/or vertigo was seen in 37.9% of the sample. Normal results in the vestibular test together with complaints pertaining to the labyrinth may be understood when the vestibular apparatus is not affected or after it has recovered^17^.

We found a significant association between a normal vestibular test and self-reported psychological complaints in 55.3% of the study sample. If vestibular symptoms result from disorders in other organs, such as neurological conditions and/or psychological disorders, dizziness may occur without functional involvement of the vestibular system^17^, which may explain the normal tests. The vestibular system is unaltered in exclusively psychogenic vertigo; thus, results of VENG are normal. The diagnostic sensitivity of VENG should also be taken into account; its results may be normal in 40% with suspected labyrinth disorders^26^, ^27^. Psychiatric conditions are frequent in patients with otoneurological complaints, especially anxiety disorders^28^. However, the presence of a psychiatric conditions does not exclude a balance disorder, which also applies inversely^28^. Psychological symptoms such as anxiety, depression, and fear, may be a cause or consequence of vertigo, or simply coexist with vertigo crises^11^, ^29^. We found no published studies on associations between vestibular test results and self-reported psychological complaints. Balance disorders and complaints of dizziness are associated with high levels of anxiety^30^. The prevalence of anxiety disorders (especially panic and agoraphobia) among patients visiting specialized centers for the treatment of balance disorders is significantly higher than that in the general population^30^, ^31^, ^32^, ^33^. Individuals with dizziness and vertigo due to vestibular disorders generally report somatic and psychic consequences, such as difficulty in concentrating, loss of memory, and fatigue. Physical insecurity because of dizziness and unbalance may result in psychic insecurity, irritability, loss of self-confidence, anxiety, depression or panic, a feeling of being out of touch with reality, and depersonalization^19^.

Mc Kenna et al.^34^ found that 42% of patients in outpatient otoneurology clinics (presenting with dizziness, tinnitus, and hearing loss) required psychological support. Jozefowicz-Korczynska et al.^35^ noted that psychological issues were often mentioned in the context of vestibular symptoms. Vestibular diseases may alter head and body alignment (patients attempt to avoid movements of the head or falls) and stability limits – the area within which individuals feel safer. There is often a mismatch between reality and the perception of stability limits in anxious individuals that fear falls; they may unnecessarily curtail their bodily movements^36^. in this context, anxiety and situational prevention approaches that characterize discomfort with movement may become a compensating strategy to avoid exposure to potentially dangerous situations^7^. The presence of vestibular dysfunction may cause a compensating increase in balance, and visual and proprioceptive sensitivity, which in turn may foster fear of heights and agoraphobia^19^.

One of the limitations of this study is that other factors not taken into account here may be related with vestibular disorders, such as use of medication (anti-inflammatory drugs, diuretics, chemotherapy, antibiotics, and psychotropic drugs), which may alter or injure the vestibular system and the cochlea; vertigo and dizziness may ensue, especially if drug dosages are high^17^. Metabolic, neurologic, cardiovascular, and degenerative conditions, infections and inflammation, trauma, otoneurologic symptoms (tinnitus and hypoacusis), and familial conditions, among others, may cause vertigo and/or dizziness^35^, ^37^. We suggest that these issues be included in future studies. Additionally, further studies on the main psychological conditions manifesting jointly with vertigo in otoneurologic patients are needed^11^.

We still suggest a longitudinal study to assess the causal relationship between anxiety and vestibular symptoms; subjects may be monitored prospectively to evaluate the development of symptoms^34^. Depression should be investigated as a psychological condition associated with stress and dizziness, and should be taken into account in the diagnosis, therapy, and progression of otoneurologic patients^12^.

## CONCLUSION

Age, sex, and the presence of absence of vertigo and/or dizziness in subjects of the study sample were not variables that had any influence on the results of vestibular testing. There was a statistically significant association between the presence of self-reported psychological complaints and normal vestibular tests. The results of VENG may be normal when vertigo is psychogenic only, as generally the vestibular system is not abnormal in these cases.
